# Does the use of modern family planning promote healthy timing and spacing of pregnancy in Dar es Salaam?

**DOI:** 10.1186/1742-4755-10-65

**Published:** 2013-12-12

**Authors:** Projestine S Muganyizi, Debora Mageta

**Affiliations:** 1 Department of Obstetrics & Gynecology, Muhimbili University of Health and Allied Sciences (MUHAS), PO Box 65117, Dar es Salaam, Tanzania

**Keywords:** Family planning, Pregnancy, Childbirth, Timing, Spacing, Tanzania

## Abstract

**Background:**

Timing, spacing and limiting of pregnancy are key outcomes of family planning (FP) whose role in promoting health of mothers and babies is evidence based. Despite the evidence, recent studies in Tanzania have reported a trend towards child birth in older age, non-adherence to standard inter-pregnancy spacing, and preference of large families in the background of a rising national contraceptive prevalence rate. We explored if the use of modern FP promotes healthy timing and spacing of pregnancy among women seeking antenatal services.

**Design:**

Analytical Cross-sectional study

**Methods:**

Women seeking antenatal services at Muhimbili National Hospital, Tanzania (August-October, 2012) were enrolled. We used a semi-structured questionnaire to obtained information from the women. Data were analyzed using SPSS version 19. Outcomes of interest were adherence to timing of first pregnancy and to inter-pregnancy spacing after normal childbirth. Use of modern FP prior to index pregnancy was the independent variable of primary interest. Bivariate and multivariate logistic regression analyses were conducted to obtain odds ratios (OR) and 95% confidence intervals (CI) as estimates risk and clinical importance respectively. Ethical approval was obtained from the Research and Publications Committee at Muhimbili University of Health and Allied Sciences.

**Results:**

In total 427 women were interviewed. Ages ranged 15–45 years, mean 29.2 (SD ± 5.1). Among all, 129 (30.2%) were primigravida, 298 (69. 8%) multigravida. Of these 298 women, 51 (17.1%) lost pregnancies preceding the index. Overall, 179 (41.9%) had ever used modern FP, 103 (24.1%) were on modern FP just prior to index pregnancy.

Non-adherence to timing was increased for primigravida (AOR = 4.5, 95% CI: 2.1-9.6) and for women older than 29 years (AOR = 7.6 95% CI: 3.8-15.2). Non-adherence to spacing was increased with loss of the immediate past pregnancy (AOR = 2.5; 95% CI: 1.3-4.7). Use of modern FP was neither associated with adherence to timing (AOR = 1.0; 95% CI: 0.5-1.9) nor spacing (AOR = 1.0; 95% CI: 0.6-1.8).

**Conclusion:**

Modern FP does not promote adherence to timing and spacing of pregnancy among women seeking antenatal services at MNH. Past obstetric experience was key to women’s decisions on spacing. There is need to promote educational messages on timing and spacing of pregnancy for healthy outcomes.

## Background

Timing, spacing and limiting of pregnancy are the three main outcomes of a planned family. For women who wish to have children, proper timing of first pregnancy and inter-pregnancy spacing are increasingly recognized as important for healthy outcomes of planned pregnancies [1-4]. In high income countries with high contraceptive prevalence there has been a trend to delay pregnancy and childbirth in favor of long inter-pregnancy intervals due to health reasons [5,6]. A similar trend has been observed in Sub-Sahara Africa but with a pattern of spacing that is little explained by such reasons [7].

Timing the first pregnancy at a young age (taken as less than 18 years) is associated with adverse effects including prematurity, low birth weight and possibly low APGAR score and maternal anemia [8-12]. Studies also indicate that becoming pregnant for the first time after 34 years of age is associated with a myriad of adverse effects to the mother and baby [13-17]. Based on current evidence, the World Health Organization (WHO) through its Technical Consultation and Scientific Review of Birth Spacing [4,18,19] recommends that after a live birth, the recommended interval before attempting the next pregnancy (i.e. inter-pregnancy interval) is at least 24 months in order to reduce the risk of adverse maternal, perinatal and infant outcomes. It further recommends that, after a miscarriage or induced abortion, the minimum interval to next pregnancy is at least six months. Concerning timing of first pregnancy the minimum recommended age at first conception is 18 years. Based on scientific evidence a concept of Healthy Timing and Spacing of Pregnancy (HTSP) emerged to advocate for adherence to WHO recommendations for better pregnancy outcomes [4,18,19] specifically recommending the use of modern Family planning (FP) methods as the best strategy to attain HTSP.

In Tanzania there has been scanty literature on HTSP. One longitudinal study in rural Tanzania indicated a high non-adherence (about 48.4%) to the recommended inter-pregnancy spacing [18]. According to Tanzania Demographic and Health Survey [20], 23% of women are mothers by 19 years of age and 16% practice shorter inter-pregnancy intervals than 2 years. In addition, there has been an increase in the national contraceptive prevalence rate from 7% in 1991/92 to the current 27% for married women 15–49 years. It is unclear if this increased use of modern contraception can be interpreted into more counseling on and adherence to HTSP. This study assessed if the use of modern FP methods was associated with adherence to HTSP among pregnant mothers seeking antenatal services at MNH.

## Methods

This analytical cross-sectional study was conducted at Muhimbili National Hospital (MNH) which is a referral and teaching hospital in Dar es Salaam, Tanzania. All women seeking antenatal services in this hospital were eligible for the study. Women who consented were consecutively selected until a calculated sample of 427 was reached between August and October, 2012. Socio-demographic, reproductive and obstetric information was obtained from antenatal cards, and from the women on one to one interviews using semi-structured questionnaires. Data were entered in EpiData version 3.1 and analyzed using IBM SPSS version 19.

Since it was important to analyze the details of women according to their prior pregnancy experiences, all women were divided into primigravida and multigravida who had ever been pregnant before. Outcomes of interest were adherence to recommended timing of first pregnancy (defined as age 18 – 30 years) and inter-pregnancy spacing after normal childbirth (24–60 months), henceforth also referred to as healthy timing and healthy spacing respectively or simply as timing and spacing. The use of modern FP methods prior to index pregnancy was the independent variable of primary interest. Other explanatory variables included maternal age, education, employment, marital status and previous pregnancy loss. Bivariate and multivariate logistic regression analyses were done to obtain odds ratios (OR) and 95% confidence intervals (CI) as estimates of risk and clinical importance of the results respectively. Ethical approval was obtained from the Research and Publications Committee at Muhimbili University of Health and Allied Sciences (MUHAS) and permission to conduct the study was granted by the Muhimbili National hospital authorities.

## Results

A total of 427 pregnant women participated, with ages ranging from 15 to 45 years and overall mean age of 29.2 (SD ±5.1 years). Most women were educated to at least secondary level (63.5%) and were in marriage (83.8%). Overall modern Family planning method use prior to the index pregnancy was 24.1% and adherence to timing of first pregnancy was 81.5% (Table 1).

**Table 1:** Characteristics of women attending antenatal services at MNH

**Characteristics**	Figures* N = 427
**Current age, Mean (±SD)yrs**	29.2(±5.1)
**Education level**	
No education	16(3.7)
Primary	140(32.8)
Secondary or above	271(63.5)
**Employment**	
Salary employment/business	310(72.6)
Housewife/other	117(27.4)
**Marital status**	
In marriage	358(83.8)
Not in marriage	69(16.2)
	
**Ever used FP method**	
No	248(58.1)
Yes	179(41.9)
**Used FP prior to index pregnancy**	
No	324(75.9)
Yes	103(24.1)
**Adherent to timing**	
No	79(18.5)
Yes	348(81.5)

*All figures are given as number (%) unless indicated otherwise.

Among all women, 129 (30.2%) were primigravida and the rest 298 (69.8%) had been pregnant before (the multigravida). The multigravida had more often used modern FP (28.2%) than the primigravida (14.7%) in the period just before the index pregnancy (p = 0.003). Non-adherence to timing was more (26.4%) among primigravida than the multigravida (15.1%), p-value = 0.006. Most primigravida (76.5%) who didn’t adhere to timing, had their first pregnancies on or beyond the age of 30 years.

Outcomes of the immediate past pregnancies for the 298 multigravida were normal childbirths (n = 189), perinatal deaths (stillbirth or baby died within one week of delivery) (n = 58) and miscarriage (n = 51), (Table 2).

**Table 2:** Obstetric characteristics of Multigravida at MNH

**Obstetric characteristics**	**Ever pregnant**
	**N =298 (%)**
**Gravidity**	
2	108(36.2)
3	100(33.6)
4+	90(30.2)
**Ever pregnancy loss**	
Yes	95(31.9)
No	203(68.1)
**Immediate past pregnancy**	
Alive	189(63.4)
Perinatal death	58(19.5)
Miscarriage	51(17.1)

### Factors associated with timing of first pregnancy

Compared to younger women, non-adherence to timing of first pregnancy was 7.6 times more likely to be reported by women who were currently older than 29 years. The risk was also more than 4 times higher for primigravida compared to the multigravida. Modern FP was not associated with healthy timing of first pregnancy (Table 3).

**Table 3:** Crude and adjusted risks for timing of the first pregnancy among women seeking antenatal services at MNH

**Characteristics**	** Adherent to timing of first pregnancy**		**Crude OR (95% CI)**	**Adjusted OR (95% CI)**
	**Yes (N = 348)**	**No = (79)**		
**Current age (yrs)**				
15-29	194(88.2)	26(11.8)	1	1
30+	154(74.4)	53(25.6)	2.6(1.5-4.3)	7.6(3.8-15.2)
**Used FP prior to index pregnancy**				
Yes	86(83.5)	17(16.5)	1	1
No	262(80.9)	62(19.1)	1.2(0.7-2.2)	1.0(0.5-1.9)
**Ever pregnancy loss**				
Yes	78(82.1)	17(17.9)	1	1
No	270(81.3)	62 (18.7)	0.7(0.4-1.2)	0.6(0.3-1.1)
**Gravidity**				
Multigravida	253(84.9)	45(15.1)	1	1
Primigravida	95(73.6)	34(26.4)	2.0(1.2-3.3)	4.5(2.1-9.6)
				
**Education level**				
No education	8(50.0)	8(50.0)	5.5(1.9-15.3)	3.2(0.7-15.0)
Primary	111(79.3)	29(20.7)	1.4(0.8-2.4)	1.3(0.7-2.6)
Secondary or above	229(84.5)	42(15.5)	1	1
				
**Employment**				
Salary employment	139(82.2)	30(17.8)	1	1
Business	121(85.8)	20(14.2)	0.8(0.4-1.4)	0.8(0.4-1.6)
Housewife/other	88(75.2)	29(24.8)	1.5(0.9-2.7)	1.4(0.7-3.1)
				
**Marital status**				
In marriage	298(83.2)	60(16.8)	1	1
Not in marriage	50(72.5)	19(27.5)	1.9(1.04-3.4)	1.8(0.8-3.9)

### Inter-pregnancy spacing

Excluding the primigravida and 51 women who had miscarried, a total of 247 women gave childbirth in the immediate past pregnancy. Out of these 247 women 125 (50.6%) conceived again within 24 and 60 months. The rest 122 (49.5%) became pregnant again either before 24 completed months (n = 50) or after a rest period of more than 60 months (n = 72). The risk for non-adherence to the recommended spacing was 2.5 times higher when the immediate past pregnancy was a perinatal death compared with normal childbirth. Other factors were not independently associated with adherence to inter-pregnancy spacing (Table 4).

**Table 4:** Crude and adjusted risks for non-adherence to spacing after a previous term birth among current antenatal clients at MNH

**Characteristics**	** Adhered to Proper Spacing**		**Crude OR (95% CI)**	**Adjusted OR (95% CI)**
	**Yes (n = 125)**	**No (n = 122)**		
**Current age (yrs)**				
19-29	16(42.1)	22(57.9)	1	1
30+	109(52.2)	100(47.8)	1.2(0.7-1.9)	1.0(0.5-1.7)
**Immediate past pregnancy loss**				
Yes	21(36.2)	37(63.8)	1	1
No	104(55.0)	85(45.0)	2.2(1.2-3.9)	2.5(1.3-4.7)
**Gravidity**				
2	49(49.5)	50(50.5)	1	1
3	39(44.8)	48(55.2)	0.8(0.5-1.5)	0.9(0.5-1.5)
4+	37(60.7)	24(39.3)	1.6(0.8-3.0)	1.8(0.9-3.7)
**Education level**				
No education	4(50.0)	4(50.0)	1	1
Primary	43(47.3)	48(52.7)	0.9(0.2-3.8)	1.0(0.2-4.6)
Secondary or above	78(52.7)	70(47.3)	1.1(0.3-4.6)	1.6(0.3-7.3)
**Employment**				
Salary employment	47(50.0)	47(50.0)	1	1
Business	43(48.9)	45(51.1)	0.9(0.6-1.6)	1.1(0.6-2.1)
Housewife/other	35(53.8)	30(46.2)	0.8(0.4-1.6)	1.6(0.7-3.4)
**Marital status**				
In marriage	113(51.1)	108(48.9)	1	1
Not in marriage	12(46.2)	14(53.8)	0.8(0.4-1.9)	0.9(0.4-2.1)
**Ever used FP method**				
Yes	67(54.5)	56(45.5)	1	
No	55(44.4)	69(55.6)	1.5(0.9-2.5)	-
**Used FP prior to index pregnancy**				
Yes	40(51.3)	38(48.7)	1	1
No	85(50.3)	84(49.7)	1.0(0.6-1.6)	1.0 (0.6-1.8)

### Inter-pregnancy spacing following a miscarriage

A miscarriage of an antecedent pregnancy was reported by 51 (17.1%) of all the 298 women who had carried a pregnancy before. In 18 (35.3%) of them, the index pregnancy was inappropriately spaced. Further analysis was compromised by a small number of cases.

## Discussion

We found a high practice of non adherence to the currently recommended timing and spacing of pregnancy among women seeking antenatal services at MNH with half of the multiparous women being non-adherent to inter-pregnancy spacing. Contrary to what would be expected, the use of modern FP was not associated with improved adherence to healthy timing and spacing of pregnancy in the study population.

This study was limited by a small sample that could not allow further analysis of women with miscarriage prior to the index pregnancy. Information on some socio-economical factors such as non-health facility delivery, residency properties, economic levels and others would have enriched our analysis but as this was a secondary analysis of unpublished data, it was not possible to obtain all that we needed from the database. Moreover, the fact that some of the information was obtained retrospectively could have heightened recall bias. Nevertheless, most of the analyzed information was on current or recent past reproductive events and was obtained directly from the women thus allowing for the flexibility to explore most desired reproductive events to the satisfaction of most of our variables.

Our finding of non-adherence to spacing in half of deliveries are in support of a recent study in rural Tanzania that revealed non-adherence to inter-pregnancy spacing in 48% of the live births conducted over 11 years of follow-up [18]. The two studies complement each other to shine light on the high magnitude of a problem that seems to be common to both rural and urban communities in Tanzania. Overwhelmingly, international studies have confirmed that too short and too long inter-pregnancy spacing predispose to poor maternal and fetal outcomes [21-27]. Scientific evidence also show that too early age (less than 18 years) and ages above 34 years at first child birth increase adverse fetal and maternal outcomes [9,15,17,28]. Despite the evidence, a fifth of women in this study did not adhere to the recommended timing of first pregnancy. The question remains, are these women informed of all these evidences? Previous studies at the same hospital have reported a progressive increase in proportion of mothers giving childbirth in older maternal ages [11], and preference to large families with the optimal family size of five children has been reported in a national survey [20]. All these have happened at a time when reports have consistently indicated improved contraceptive prevalence rate in Tanzania [20]. It was therefore justifiable to explore whether or not the use of modern FP promoted adherence to HTSP among users.

Ideally counseling for FP should include evidence based information on sexual health including healthy timing and spacing of pregnancies [29]. Since modern FP is the best known strategy to control fertility and achieve HTSP [28], increased FP method use should improve adherence to HTSP through increased exposure to HTSP messages and the contraceptive benefits of the methods per se. Contrary to this contention, we found no association between FP use and adherence to HTSP in the current study. Our findings suggest that modern FP in Tanzania is partly used to fulfill unhealthy reproductive preferences. We demonstrated that the risk for non-adherence to spacing of the index pregnancy was three times more when the immediate past pregnancy ended in child death than when it ended in a live baby independent of parity and other socio-economical and reproductive factors. This confirms that there are other factors that are given more priority when considering inter-pregnancy spacing than just healthy outcomes of pregnancy. These findings are in support of Moultrie et al. who reported a different pattern of childbirth spacing in some Sub-Sahara African countries which was not explainable by the differences in parity and maternal age. According to Moultrie et al., the pattern seen in high income countries was largely explained by known health reasons such as need for breast feeding and recovery from nutritional losses between pregnancies [7]. A low impact of FP on inter-pregnancy spacing across countries has also been recently documented by Stover and Ross [28].

Education level of the participants to this study was positively associated with adherence to the recommended age at first pregnancy but not to spacing. Since some information on timing and spacing for the women who had been pregnant before was retrospective, current education level was unlikely to have had direct influence on what happened in the past. However, current attainment of high level education was considered a valid indicator of a longer time spent in school which could be interpreted into longer postponement of risks of pregnancy as compared with women who did not go to school or who spent a short time in studies. Due to these reasons the importance of education in timing of first pregnancy was completely lost after controlling for age and parity. Overall, the risk of non-adherence to timing of first pregnancy was more than twice for primigravida compared with the multigravida. Primigravidity could be viewed as a proxy to the young generation as supported by their substantially younger mean current age compared to multigravida. Their tendency to become pregnant at older ages could be a reflection of a cohort effect of the observed global trend to give birth at older ages even for those who did not spent a substantially long time in school [7]. Nevertheless, the above relationship can only be partial since maternal age also emerged independently as predictor for non-adherence to timing of pregnancy, thus supporting Tanzania’s Demographic and Heath Survey data that indicate a substantial and progressive decrease in childbirth among teenagers in favor of birth in older reproductive ages [20].

Our results have questioned the effectiveness of current FP services in promoting HTSP in Dar es Salaam. User attitude to timing and spacing is apparently a major underlying factor for non-adherence. Polices should put in place effective educational messages on HTSP as part and parcel of routine FP services and the education should respond to social and reproductive concerns of the users. Further in-depth studies are needed to understand the reasons underlying non-adherence to HTSP in Tanzania.

## Conclusions

Adherence to the recommended timing and spacing of pregnancy and childbirth is low among women who seek antenatal services at MNH. The practice of modern FP does not promote adherence to both timing and inter-pregnancy spacing. Past obstetric events seem to be an important factor in predicting decisions on adherence to spacing of pregnancy.

## Competing interests

The authors declare that they have no competing interests.

## Authors’ contributions

Both authors contributed to the planning and design of the study. DM did data collection. PS did secondary analysis of the data and manuscript writing. Both authors read and approved the final draft of the manuscript.

## Authors’ information

PS (MD, MMed, PhD) is a consultant Obstetrician/ Gynecologist and senior lecturer at MUHAS. DM is obstetrician/Gynecologist in the Department of Obstetrics & Gynecology, MUHAS.

## References

[B1] Zhu BP Le T Effect of interpregnancy interval on infant low birth weight: a retrospective cohort study using the Michigan Maternally Linked Birth Database Matern Child Health J 2003 10 3 169 178 10.1023/A:1025184304391 14509412

[B2] Yigzaw M Enquselassie F Birth spacing and risk of child mortality at Kalu district South Wollo Zone of Amhara region, Ethiopia Ethiop Med J 2010 10 2 105 115 20608014

[B3] Luo ZC Karlberg J Timing of birth and infant and early neonatal mortality in Sweden 1973–95: longitudinal birth register study Bmj 2001 10 7325 1327 1330 10.1136/bmj.323.7325.1327 11739216 PMC60669

[B4] Lane C Joof YM Hassan AA Pryor S Promoting healthy timing and spacing of pregnancy with young married women in Northern Nigeria: a short report Afr J Reprod Health 2012 10 2 263 269 22916558

[B5] Ventura SJ Hamilton BE U.S. teenage birth rate resumes decline NCHS Data Brief 2011 10 1 8 21592421

[B6] Ventura SJ Mathews TJ Hamilton BE Teenage births in the United States: state trends, 1991–2000, an update Natl Vital Stat Rep 2002 10 9 1 4 12060957

[B7] Moultrie TA Sayi TS Timaeus IM Birth intervals, postponement, and fertility decline in Africa: a new type of transition? Popul Stud (Camb) 2012 10 3 241 258 10.1080/00324728.2012.701660 22891624

[B8] Muganyizi PS Balandya B Pregnancy outcomes in the extremes of reproductive age: a seven-year experience in Tanzania Open J Obstet Gynecol 2013 10 51 57

[B9] Gibbs CM Wendt A Peters S Hogue CJ The impact of early age at first childbirth on maternal and infant health Paediatr Perinat Epidemiol 2012 10 Suppl 1 259 284 22742615 10.1111/j.1365-3016.2012.01290.x PMC4562289

[B10] Chantrapanichkul P Chawanpaiboon S Adverse pregnancy outcomes in cases involving extremely young maternal age Int J Gynaecol Obstet 2013 10 2 160 164 10.1016/j.ijgo.2012.08.024 23182803

[B11] Muganyizi PS Kidanto HL Impact of change in maternal age composition on the incidence of Caesarean section and low birth weight: analysis of delivery records at a tertiary hospital in Tanzania, 1999–2005 BMC Pregnancy Childbirth 2009 10 30 10.1186/1471-2393-9-30 19622146 PMC2718860

[B12] Muganyizi PS Kidanto H Sickle cell disease in pregnancy: trend and pregnancy outcomes at a tertiary hospital in Tanzania PLoS One 2013 10 2 e56541 10.1371/journal.pone.0056541 23418582 PMC3572068

[B13] Carolan MC Davey MA Biro M Kealy M Very advanced maternal age and morbidity in Victoria, Australia: a population based study BMC Pregnancy Childbirth 2013 10 1 80 10.1186/1471-2393-13-80 23537152 PMC3637179

[B14] de Weger FJ Hukkelhoven CW Serroyen J te Velde ER Smits LJ Advanced maternal age, short interpregnancy interval, and perinatal outcome Am J Obstet Gynecol 2011 10 5 421 e421-429 21288503 10.1016/j.ajog.2010.12.008

[B15] Kenny LC Lavender T McNamee R O'Neill SM Mills T Khashan AS Advanced maternal age and adverse pregnancy outcome: evidence from a large contemporary cohort PLoS One 2013 10 2 e56583 10.1371/journal.pone.0056583 23437176 PMC3577849

[B16] Lisonkova S Pare E Joseph K Does advanced maternal age confer a survival advantage to infants born at early gestation? BMC Pregnancy Childbirth 2013 10 87 10.1186/1471-2393-13-87 23566294 PMC3637212

[B17] Balayla J Azoulay L Assayag J Benjamin A Abenhaim HA Effect of maternal age on the risk of stillbirth: a population-based cohort study on 37 million births in the United States Am J Perinatol 2011 10 8 643 650 10.1055/s-0031-1276739 21544772

[B18] Exavery A Mrema S Shamte A Levels and correlates of non-adherence to WHO recommended inter-birth intervals in Rufiji, Tanzania BMC Pregnancy Childbirth 2012 10 152 10.1186/1471-2393-12-152 23237623 PMC3573999

[B19] WHO Report of a WHO technical consultation on birth spacing 2007 Geneva,Switzerland: WHO

[B20] NBoSaIM Tanzania demographic and health survey 2010 2011 Dar e Salaam Tanzania: NBS and ICF Macro

[B21] Conde-Agudelo A Belizan JM Breman R Brockman SC Rosas-Bermudez A Effect of the interpregnancy interval after an abortion on maternal and perinatal health in Latin America Int J Gynaecol Obstet 2005 10 Suppl 1 S34 40 15820366 10.1016/j.ijgo.2004.08.003

[B22] Conde-Agudelo A Rosas-Bermudez A Castano F Norton MH Effects of birth spacing on maternal, perinatal, infant, and child health: a systematic review of causal mechanisms Stud Fam Plann 2012 10 2 93 114 10.1111/j.1728-4465.2012.00308.x 23175949

[B23] Conde-Agudelo A Rosas-Bermudez A Kafury-Goeta AC Birth spacing and risk of adverse perinatal outcomes: a meta-analysis JAMA 2006 10 15 1809 1823 10.1001/jama.295.15.1809 16622143

[B24] Conde-Agudelo A Rosas-Bermudez A Kafury-Goeta AC Effects of birth spacing on maternal health: a systematic review Am J Obstet Gynecol 2007 10 4 297 308 10.1016/j.ajog.2006.05.055 17403398

[B25] Dewey KG Cohen RJ Does birth spacing affect maternal or child nutritional status? A systematic literature review Matern Child Nutr 2007 10 3 151 173 10.1111/j.1740-8709.2007.00092.x 17539885 PMC6860904

[B26] Goncalves SD Moultrie TA Short preceding birth intervals and child mortality in Mozambique Afr J Reprod Health 2012 10 4 29 42 23444541

[B27] Orji EO Shittu AS Makinde ON Sule SS Effect of prolonged birth spacing on maternal and perinatal outcome East Afr Med J 2004 10 8 388 391 15622931 10.4314/eamj.v81i8.9198

[B28] Stover J Ross J Changes in the distribution of high-risk births associated with changes in contraceptive prevalence BMC Public Health 2013 10 Suppl 3 S4 10.1186/1471-2458-13-S3-S4 24564577 PMC3847521

[B29] Krychman M Counselling: a life course approach Gynaecol Forum 2013 10 2 13 16

